# 

**Published:** 1906-06-16

**Authors:** 


					THE HOSPITAL.
_No. 1,029. 'Vol. XL. [SfiSj?,*) , Satukday,
June 16th, 1906.
[Subscription Terms
see p. 202,
] PRICE THREEPENCE

				

